# Cross-reactive immune responses to monkeypox virus induced by MVA vaccination in mice

**DOI:** 10.1186/s12985-023-02085-0

**Published:** 2023-06-19

**Authors:** Feixia Gao, Cheng He, Min Liu, Ping Yuan, Shihua Tian, Mei Zheng, Linya Zhang, Xu Zhou, Fangjingwei Xu, Jian Luo, Xiuling Li

**Affiliations:** 1 Shanghai Institute of Biological Products, Shanghai, China; 2 China National Biotec Group, Beijing, China

**Keywords:** MVA, Monkeypox virus, Protective antigens, Humoral immune response, Cellular immune response

## Abstract

Mpox (monkeypox) infection cases increased recently in non-Mpox outbreak areas, potentially causing an international threat. The desire to defend against a potential outbreak has led to renewed efforts to develop Mpox vaccines. In this report, mice were immunized with various doses of modified vaccinia virus Ankara (MVA) to evaluate the cross-reactive immune response of MVA immunization against protective antigens of the current monkeypox virus. We demonstrated that MVA induced specific antibodies against protective antigens (A29, A35, B6, M1, H3, and I1), mediating the neutralization abilities against the MVA and the monkeypox virus (MPXV). Moreover, recombinant protective antigens of the MPXV elicited cross-binding and cross-neutralizing activities for MVA. Hence, the MVA induced cross-reactive immune responses, which may guide future efforts to develop vaccines against the recent MPXV. Notably, compared to the other protective antigens, the predominant A29 and M1 antigens mediated higher cross-neutralizing immune responses against the MVA, which could serve as antigen targets for novel orthologous orthopoxvirus vaccine.

## Introduction

Monkeypox (Mpox) is a zoonotic disease caused by the monkeypox virus (MPXV), which leads to a smallpox-like disease in humans. The first case of Mpox in humans was identified in 1970 in the Democratic Republic of Congo, then the virus became more widespread within the African continent [1]. In early May 2022, the resurgence of Mpox in non-African nations posed a potential threat to humans, leading the World Health Organization (WHO) to declare the Mpox outbreak a global health emergency on July 23. Within the following months, thousands more cases were identified in over 110 countries and regions, including 112 deaths (https://www.cdc.gov/poxvirus/monkeypox/response/2022/world-map.html).

Orthopoxvirus of any one species may confer cross-reactivity [2]. Due to the antigenic similarity between vaccinia virus (VACV) and MPXV, vaccination with smallpox vaccines is considered one of the measures to control the Mpox outbreak [3]. Two smallpox vaccines approved for use in the United States and Europe include the second-generation vaccine, ACAM2000 (replication-competent live vaccinia virus), which cannot be used in the immunocompromised, and the third-generation modified vaccinia virus Ankara Bavarian Nordic (MVA-BN) vaccine (brand names JYNNEOS, IMVAMUNE, IMVANEX), which contains a replication-deficient live vaccinia virus and is safe for the immunocompromised [4]. The MVA vaccine is a highly attenuated virus that was used as a vaccine against human smallpox in Turkey and Germany in the 1970s. MVA vaccination offers protection against lethal orthopoxvirus, including MPXV in non-human primates, rabbitpox virus in rabbits, and VACV in mice [5–8]. Phase II clinical trials found that MVA-BN was safe and well-tolerated in immunocompromised [9, 10]. Smallpox vaccination was 85% protective against MPXV, according to the U.S. Centers for Disease Control and Prevention and the World Health Organization [11, 12]. In the latest report, the average differences of 50 single nucleotide polymorphisms existed in the current MPXV and related to 2018–2019, which may indicate an accelerated evolution of MPXV [13]. So far, ACAM2000 and IMVAMUNE are recommended for persons at risk for Mpox, and preliminary vaccine efficacy data on JYNNEOS used in the USA are about 69% against medically attended Mpox disease during the recent outbreak(https://www.cdc.gov/poxvirus/mpox/cases-data/JYNNEOS-vaccine-effectiveness.html). Hence, the MVA might still be effective on current outbreak strains and in preventing Mpox diseases.

MPXV is a double-stranded DNA virus of the Orthopoxvirus genus in the Poxviridae family, with a genome size of about 197 kb and encoding at least 190 non-overlapping open reading frames [4]. The virus exists in two antigenically distinct forms: mature virion (MV) and enveloped virion (EV). MV is assembled in the cytoplasm of virus-infected cells and is responsible for viral infection transmission between hosts. At the same time, EV is responsible for direct intercellular transmission and remote virus transmission in hosts [14–16]. MV surface proteins L1, A27, A17, H3, and D8, the targets for neutralizing antibodies, could mediate the MV adsorption on the cell surface and play a role in viral infection through the entry and fusion process. A27 participates in virus-cell attachment, virus-cell fusion, and viral release from cells, H3 binds to cell surface molecules, L1 and A28 are required for viral entry into cells, and I1 mediates cell membrane fusion and MV nuclear invasion [17–19]. The exogenous trans-membrane protein B5 on EV particles is associated with MV encapsulation, EV morphogenesis, and viral release from cells [20–22]. A33 is involved in mediating EV membrane lysis, complement-mediated lysis, or T cell response [23]. Antibodies targeting MV surface proteins A27, L1, H3, D8, A28, A13, A17, and EV surface proteins B5 and A33 effectively cross-neutralized VACV, cowpox virus (CPXV), MPXV and variola virus (VARV) [24]. These protective antigens (PAs) of MPXV may be used to evaluate the immunogenicity of the MVA against the current Mpox.

To investigate whether MVA immunization can elicit antibodies that cross-react with orthologous PAs of MPXV, mice were immunized with different doses of MVA. Here, we reported that MVA could elicit specific IgG antibodies against MPXV PAs, neutralizing antibodies against the MVA virus and the current MPXV, and activate cellular immune responses. Importantly, the PAs of MPXV also were cross-reactive with the MVA virus, by activating humoral immune responses, which could serve as major antigen targets for the orthologous orthopoxvirus vaccine. In this study, we elucidated that MVA could effectively cross-react with MPXV , implying that this traditional smallpox vaccine could still be valuable in the Mpox prevention, and worth further development to control the outbreak.

## Materials and methods

### Cells and viruses

Primary chicken embryo fibroblast cells (CEF) and BHK-21 cells (ATCC CCL-10) or Vero cells (ATCC CRL-1587) were cultured in 199 medium (Gibco, No.11150059) and MEM medium (Gibco, No.10370070) with 10% fetal bovine serum (Gibco, No.25200) at 5% CO_2_ and 37 ℃.

Modified vaccinia virus Ankara (MVA, GenBank: U94848.1) was obtained from ATCC (VR-1508). MVA was propagated on CEF for 72 h, then CEF monolayers were harvested. Cells were pelleted by spinning and sonicated to break up clumps. After sucrose gradient centrifugation, the purified virus supernatants were filtered through a 0.45 µM filter membrane, and aliquots of the virus were stored at -70 ℃ [25, 26]. MVA titer was determined by TCID_50_ assay in BHK-21 cells [27].

### Animal immunization with MVA

The protocol for the animal study (Protocol Number:2022005) was approved by the laboratory animal management committee and the laboratory animal ethics and welfare protection group of the Shanghai Institute of Biological Products.

Six-week-old BALB/c mice (n = 5 per group) were intramuscularly vaccinated twice at day 0 and day 28 with MVA (50 µL/dose) at concentrations of 10^5^ TCID_50_/mL, 10^6^ TCID_50_/mL, and 10^7^ TCID_50_/mL, respectively; control mice were mock-vaccinated (PBS, 50 µL per dose). Serum samples were collected on days 14, 28, 42, and 70, and stored at -20 ℃ for serological analysis.

### Animal immunization with MPXV protective antigens

Six-week-old BALB/c mice (n = 5 per group) were intramuscularly vaccinated twice at day 0 and day 21 with 10 µg recombinant MPXV PAs (A29, M1, H3, I1, A35, and B6 protein, expressed in Chinese hamster ovary cells by Shanghai Institute of Biological Products) or 60 µg Mixed PAs containing 50 µL AddaVax ™ (InvivoGen, San Diego, CA, USA) adjuvant or non-adjuvant. Inoculations of corresponding amounts of PBS were used as controls (mock vaccine). Serum samples were collected on day 35 and stored at -20 ℃.

### Enzyme-linked immunosorbent assay (ELISA)

To determine total IgG antibody titers in immunized mouse sera, microtiter plates were coated with 100 µL/well of each MPXV PA (1 µg/mL, A29, M1, H3, I1, A35, and B6, respectively) or inactivated MVA virus (2 µg/mL) at 4 ℃ overnight. Plates were incubated for 1 h at 37 °C with blocking buffer (5% albumin bovine serum in PBS with 0.5% Tween 20). Mouse sera were 2-fold serially diluted in blocking buffer and incubated at 37 °C for 1 h, then incubated with rabbit anti-mouse IgG peroxidase-conjugated antibody at 37 °C for 1 h. TMB was added, following the addition of 2 M H_2_SO_4_, to stop the reaction. The means + 2 × SD (standard deviations) of the mock group (PBS group) were used for the antibody-positive cut‐off values set. End-point titers were determined as the highest dilution with an absorbance value greater than the cut‐off values [28].

### Plaque reduction neutralization test (PRNT)

The titers of neutralizing anti-MVA virus antibodies were determined by plaque reduction neutralization test. Briefly, each serum was diluted 2-fold with DMEM. An equal volume of MVA virus at a concentration of 100 TCID_50_ was added and incubated at 37 °C for 1 h, then the mixture samples were added into BHK-21 cells with repeated eight wells for each sample. Virus plaques (cytopathic effect) were visualized after incubation at 37 ℃ for 72 h. The percent neutralization was calculated relative to the number of wells with plaques. The titers of the sera were determined by the reciprocal of the highest dilution that gave 50% plaque reduction [29, 30]. Additionally, diluted sera were mixed with 600 pfu of MPXV strain (hMpxV/Hong Kong/HKU-220914-001/2022) and adsorbed onto Vero cells for further neutralization test against MPXV.

### Multi-Analyte flow assay

A multi-analyte flow assay was used to detect the cellular immune response induced by MVA. Spleen lymphocytes at day 70 postimmunization were isolated and inoculated on 48-well plates according to the density of 1×10^7^ cells/mL by stimulating with 10 µg/mL MPXV PAs (A29, M1, H3, I1, A35, and B6, respectively). The cell’s supernatants were collected after incubation at 37 ℃ for 48–72 h. The levels of secreted cytokines, including IFN-γ, TNF-α, IL-2, IL-4, IL-6, IL-17A, and IL-22, were quantified using a custom LEGENDplex ™ Mouse Multi-Analyte Flow Assay kit (Cat # 741044; BioLegend). Results were expressed as pg/mL of timed splenocytes culture supernatants in each sample.

### Statistics

Analyze data in GraphPad Prism 9.0 software. T-test was used to analyze the comparison between the two groups, and One-way ANOVA was used for multiple comparisons. Significance levels were set at a *P* value of 0.05.

## Results

### Gene homology analysis of the protective antigens between MVA and monkeypox virus

MVA and MPXV belong to Orthopoxvirus genus, which share a significant homology in the central conserved region. The amino acid sequences of MVA (GenBank: U94848.1) PAs (*A29, A35, B6, M1, H3* and *I1*) were compared to the entries of current MPXV (GenBank: ON563414.3) sequence databases (Table 1). Sequence alignments revealed that the PAs of MVA and MPXV homologs shared the same initiation codons with at least 93% amino acid identity, moreover, with 0% gap accessibility. M1 and 11 proteins were highly conserved, at least 98% identical. Following was B6 protein, with 96.2% identity. A35 and H3 proteins were at least 94% identical, while A29 protein was 93.6%.

**Table 1:** Amino acid alignment of antigen orthologues between MVA and monkeypox virus

Antigen		Virion form	kDa	Blast Amino acid		Functions in virus infection and replication
MVA	MPXV					
Accession No. U94848.1	Accession No. ON563414.3			Similarity(%)	Gap(%)	
*A27*	*A29*	MV	12.5	93.6 (103/110)	0.0 (0/110)	Fusion protein, virus-cell attachment, virus-cell fusion, and virus release from cells
*A33*	*A35*	EV	20.6	94.5 (171/181)	0.0 (0/181)	Glycoprotein, an integral component of the membrane, lyses the EV membrane
*B5*	*B6*	EV	35.1	96.2 (305/317)	0.0 (0/317)	Complement control protein, MV wrapping, EV morphogenesis, and release of the virus from the cell
*L1*	*M1*	MV	27.3	98.8 (247/250)	0.0 (0/250)	Myristylated MV surface membrane protein, cellular entry
*H3*	*H3*	MV	37.5	94.4 (306/324)	0.0 (0/324)	Immunodominant MV protein, attachment of poxviruses to cells, viral morphogenesis
*I1*	*I1*	MV	35.9	98.7 (308/312)	0.0 (0/312)	DNA (telomere)-binding core protein

EV: enveloped virion; MV: mature virion.

### MVA-induced humoral immune responses against MPXV protective antigens

To determine the humoral immune response against MPXV PAs, mice were immunized twice with various virus concentration, which is 10^5^ TCID_50_/mL (10^5^), 10^6^ TCID_50_/mL (10^6^), and 10^7^ TCID_50_/mL (10^7^) for MVA, and sera were collected on days 14, 28, 42, and 70 postimmunization for further serological detection.

**Fig. 1:**
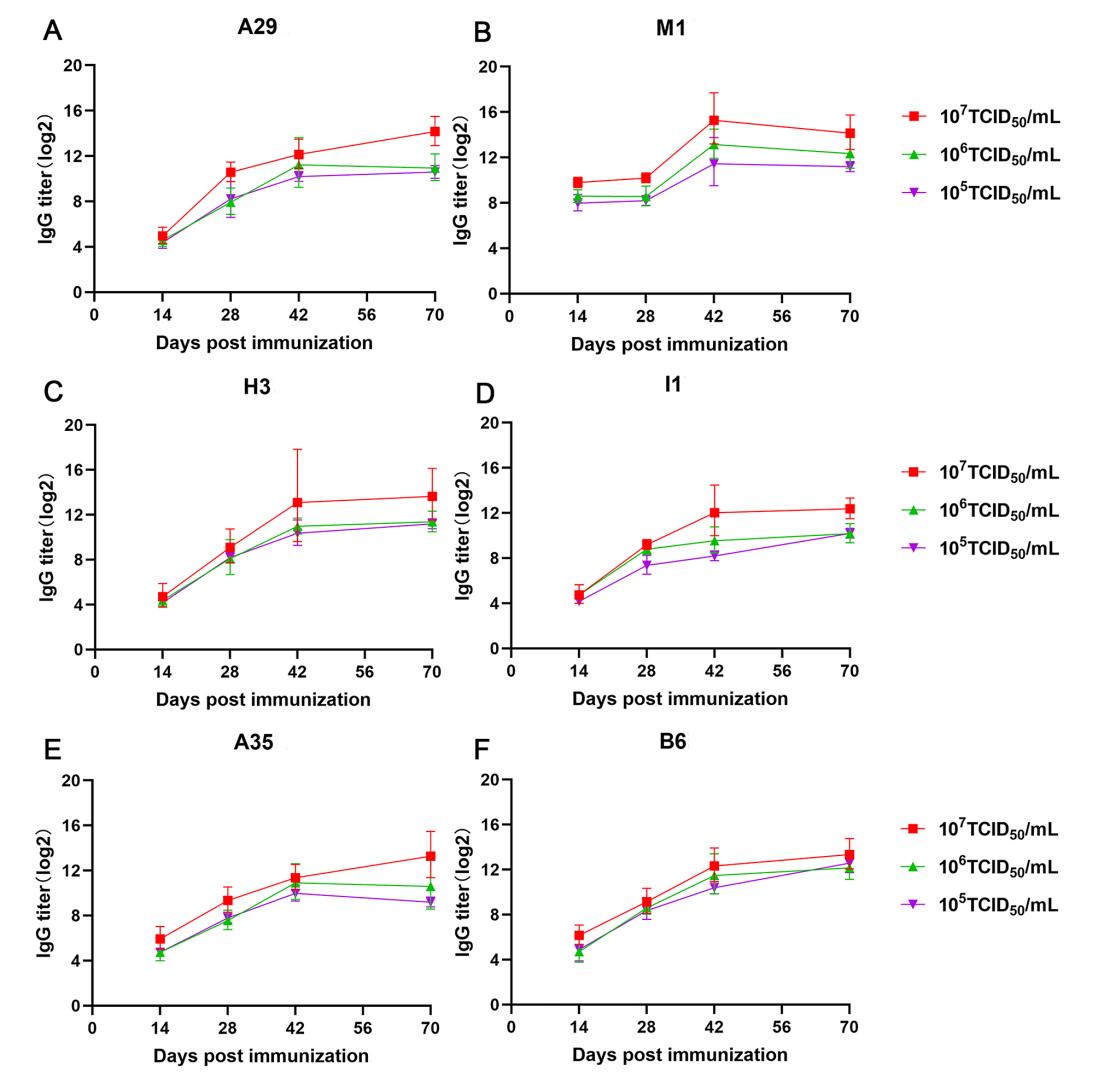
MVA-induced specific IgG antibodies against different MPXV PAs. BALB/c mice (n = 5) were immunized twice with different MVA concentrations. Sera from vaccinated mice on days 14, 28, 42, and 70 postimmunization were tested for MPXV-specific antibodies using (**A**) A29-specific ELISA; (**B**) M1-specific ELISA; (**C**) H3-specific ELISA; (**D**) I1-specific ELISA; (**E**) A35-specific ELISA and (**F**) B6-specific ELISA. A 1 µg/mL of each PA was used for plate coating.

The antibody response against MPXV PAs in the immunized serum was tested using ELISA assay (Fig. 1). After the initial priming vaccination, antibody responses were detected against each PA in most animals, while no significant differences in the titers among groups with different doses of MVA vaccine were observed. Except for M1, responses in groups receiving MVA on day 28 were significantly higher than those on day 14 after the prime (*P* < 0.05). Indeed, two weeks after the boost immunization (day 42), the anamnestic responses markedly increased for each PA, while the response to each PA was not significantly enhanced on day 70 compared to those on day 42. These results indicated that MVA could induce specific IgG antibodies against A29, M1, H3, I1, A35, and B6, and the antibody response in 10^7^ TCID_50_/mL MVA immunized group was higher than other groups, with a dose-dependent manner.

**Fig. 2:**
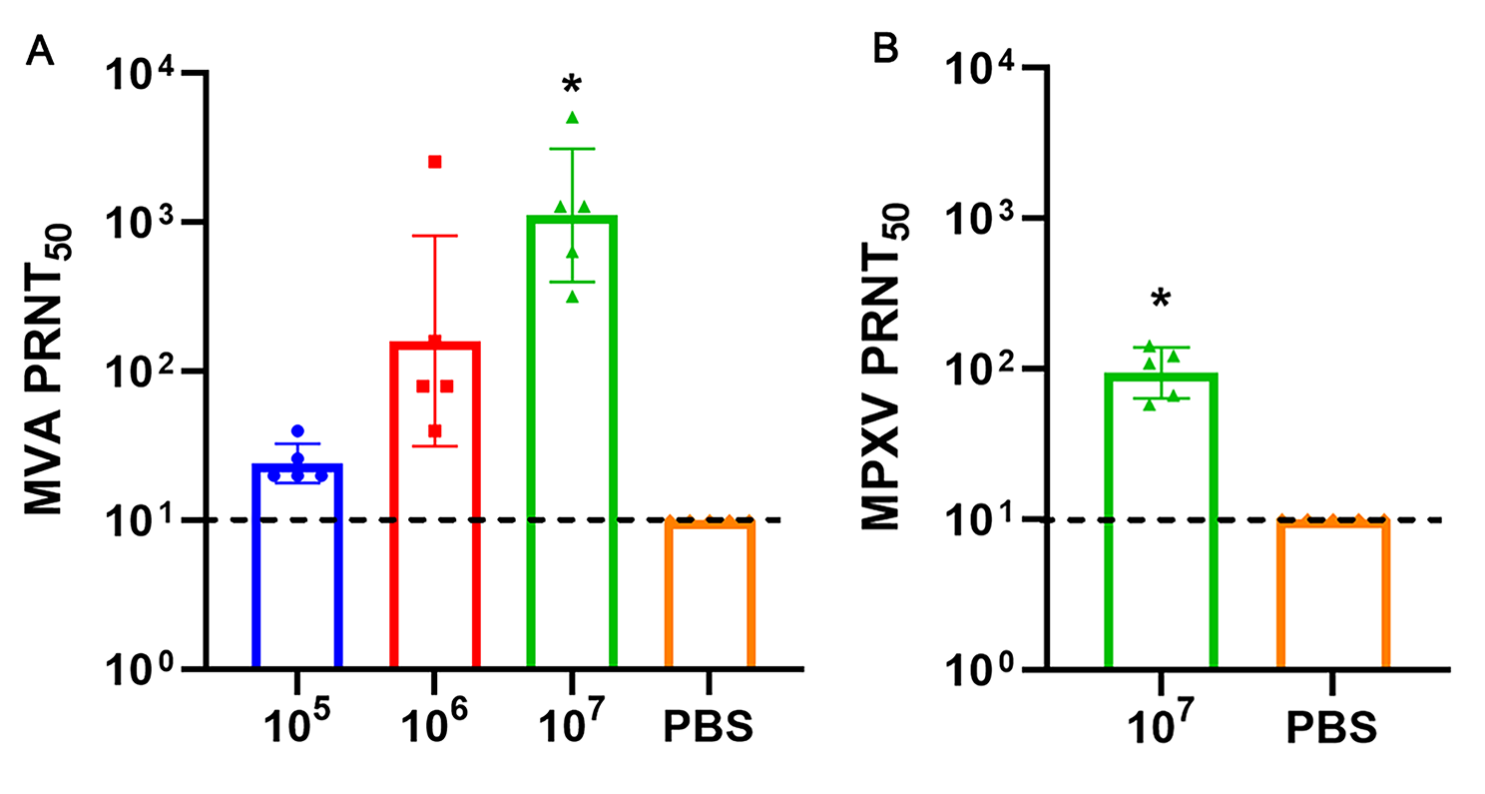
MVA-induced neutralizing antibodies against MVA or MPXV. BALB/c mice (n = 5) were immunized twice with different MVA concentrations. (**A**) On day 42 postimmunization, the neutralizing antibody titers against MVA virus induced by MVA were determined by PRNT with 100 TCID_50_ MVA virus on BHK-21 cells. (**B**) On day 42 postimmunization, the neutralizing antibody titers against MPXV induced by MVA concentration of 10^7^ TCID_50_/mL were determined by PRNT with 600 pfu MPXV on Vero cells. Comparing the vaccination effect among MVA and PBS groups, the asterisk * indicates a significant difference (*P* < 0.05) detected by one-way ANOVA or T-test analysis.

Neutralizing antibodies have been used to evaluate the immunogenicity of vaccines. We investigated the functionality of the antibody responses by performing PRNT to measure neutralizing antibodies against MVA or MPXV (Fig. 2). After the boost immunization, all groups receiving the MVA induced neutralizing antibody responses against the MVA in a dose-dependent manner. Animals vaccinated with an MVA concentration of 10^7^ TCID_50_/mL had significantly higher responses compared to the PBS group (GMT = 1114 versus GMT = 10, *P* < 0.05)(Fig. 2A). Importantly, we also found that vaccination of 10^7^ TCID_50_/mL MVA was sufficient to induce neutralizing antibodies against the current MPXV (GMT = 94.3), which made the difference over the PBS group (*P* < 0.05) (Fig. 2B).

Overall, our findings indicated that MVA significantly enhanced the immune responses against A29, M1, H3, I1, A35, and B6 antigens, along with high neutralizing antibodies against MVA and MPXV. Considering the highly conserved PAs between MVA and MPXV, the neutralizing activity elicited by vaccination with MVA might also cross-react with MPXV.

### Cellular immune responses against MPXV protective antigens induced by MVA

The cellular immune response plays a key role in the rapidly protective immunization of vaccines. To assess cellular immune response, the overall T cell responses were measured by multi-analyte flow assay following specific stimulation with each A29, M1, H3, I1, A35, and B6 protein used as immunogens.

**Fig. 3:**
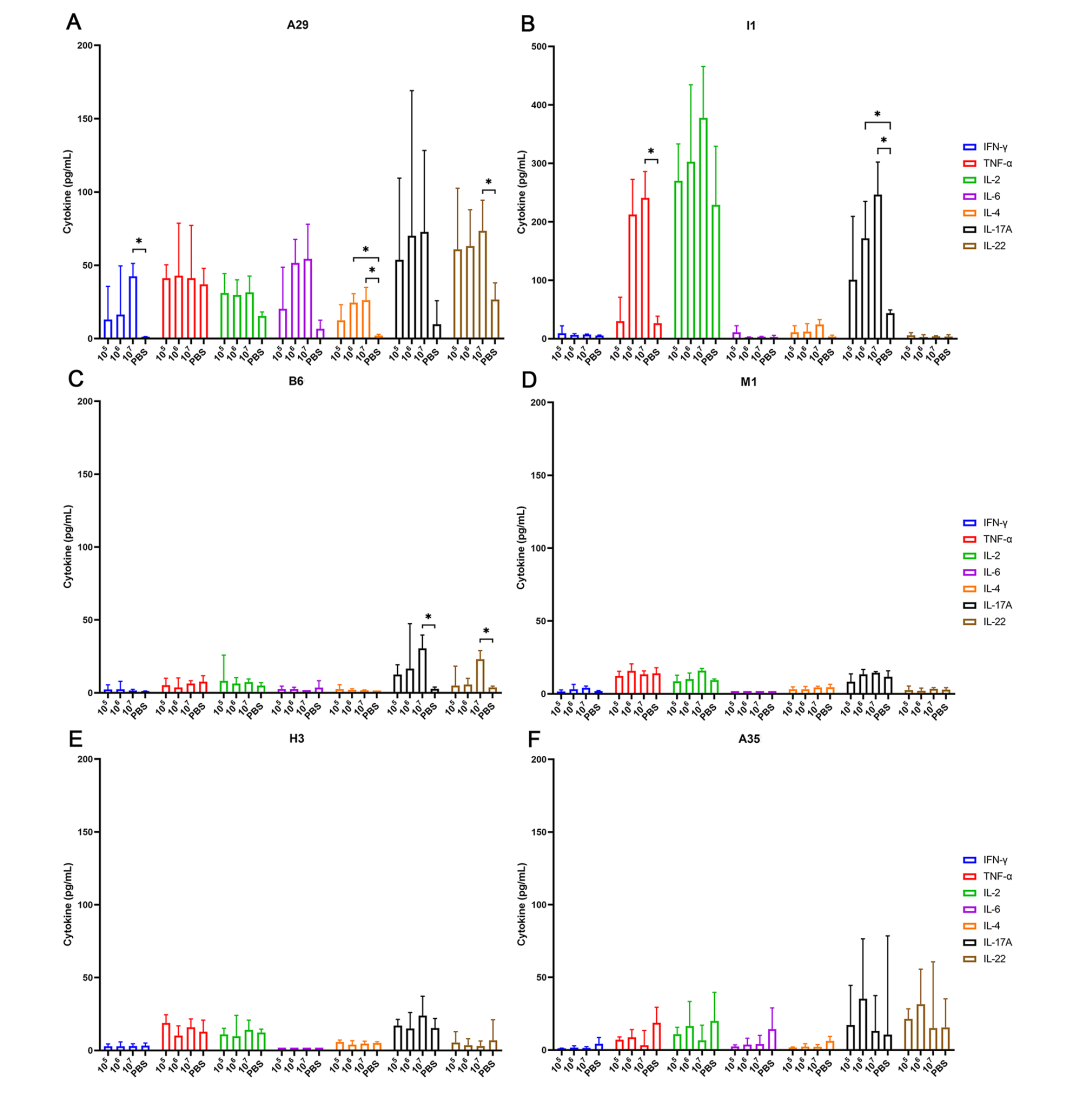
MVA-induced cellular immune responses stimulated by MPXV PAs. BALB/c mice (n = 5) were immunized twice with different MVA concentrations. On day 70 postimmunization, the cellular immune responses to different PAs induced by MVA were detected by multi-analyte flow assay, (**A**) A29, (**B**) I1, (**C**) B6, (**D**) M1, (**E**) H3, and (**F**) A35. Comparing the levels of various cytokines among MVA and PBS groups, the asterisk * indicates a significant difference (*P* < 0.05) detected by one-way ANOVA.

Our results showed that IFN-γ, TNF-α, IL-2, IL-6, IL-4, IL-17A, and IL-22 could be measured in the lymphocytes of MVA vaccinated groups stimulated by A29 protein. The 10^7^ TCID_50_/mL MVA immunized group showed a significant increase of IFN-γ and IL-22 cytokines compared to the PBS group (*P* < 0.05), showing a dose-dependent manner.The 10^6^ and 10^7^ TCID_50_/mL immunization groups could produce reasonable levels of A29-specific IL-4 cytokine, which were statistically different from the PBS group (*P* < 0.05). Except for TNF-α cytokine, MVA immunized groups showed an increase of IL-2/IL-6/IL-17A cytokines, but no significant differences were observed when compared to the PBS group (Fig. 3A).

Moreover, the inductions of TNF-α, IL-2, and IL-17A cytokines stimulated by I1 protein could be detected in MVA-vaccinated groups (Fig. 3B). Compared to the PBS group, the 10^7^ TCID_50_/mL MVA immunized group could induce higher levels of TNF-α and IL-17A cytokines (*P* < 0.05), and each immunization group also induced higher levels of IL-2. However, there was no statistical difference among the groups. In addition, the 10^7^ TCID_50_/mL MVA immunized group showed a significant increase of IL-17A and IL-22 cytokines stimulated by B6 protein compared to the PBS group (Fig. 3C). However, we found only slight increases of IFN-γ, TNF-α, IL-2, IL-6, IL-4, IL-17A, and IL-22 cytokines stimulated by M1, H3, and A35 proteins in the immunized groups, and no statistical differences among these groups were observed (Fig. 3D, E and F).

Together these findings demonstrated that MVA could elicit a humoral immune response against MPXV PAs, and cellular immune responses specifically recognizing the A29, I1, and B6 of MPXV PAs. Therefore, the protective immune responses induced by MVA might protect against the invading MPXV infection.

### Humoral immune responses induced by various recombinant protective antigens of the monkeypox virus

To assess whether recombinant MPXV protective antigens, expressed in Chinese hamster ovary cells, can produce cross-neutralizing antibodies against the MVA, and further verify the immunogenicity of MVA against MPXV, the humoral immune responses induced by recombinant PAs were determined. Mice were immunized with various PAs or mixed PAs with AddaVax or without adjuvant, and an MVA concentration of 10^7^ TCID_50_/mL as a positive control. Sera were collected on day 35 postimmunization to evaluate antibody responses.

**Fig. 4:**
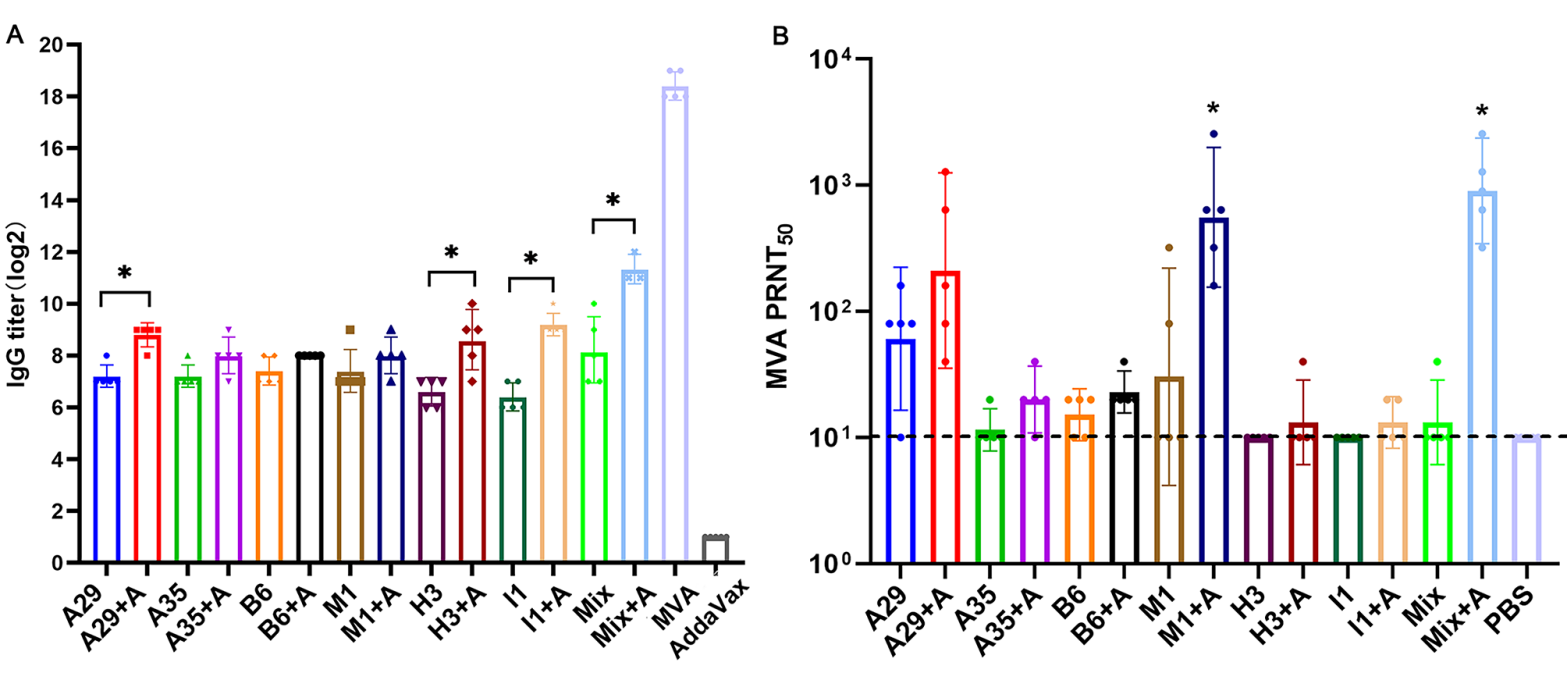
Humoral immune responses induced by various protective antigens of MPXV. BALB/c mice (n = 5) were immunized twice with different PAs or mixed PAs with or without AddaVax adjuvant, and 10^7^ TCID_50_/mL MVA as a positive control. On day 35 postimmunization, sera were collected to determine humoral immune responses induced by various MPXV PAs. (**A**) The titers of IgG antibody against MVA were evaluated by ELISA assay. MVA strain was used as ELISA antigen. Comparing the levels of IgG antibody among the adjuvanted antigen group and the non-adjuvanted antigen group, the asterisk * indicates a significant difference (*P* < 0.05) detected by T-test analysis. (**B**) MVA-neutralizing antibody titers were determined by PRNT. Comparing the vaccination effect among MPXV PAs and PBS groups, the asterisk * indicates a significant difference (*P* < 0.05) detected by one-way ANOVA.

After the boost vaccination, mice vaccinated with various PAs with or without adjuvant could produce antibody responses to MVA virus, as measured by immunogen-specific ELISA (Fig. 4A). The titers of IgG antibody were significantly higher (*P* < 0.05) in the group receiving AddaVax-adjuvanted mixed PAs (Mix + A, GMT = 2^11^) compared to those not receiving adjuvant, which was lower than the positive control (MVA, GMT = 2^18.4^). The antibody responses elicited by A29, I1, and H3 antigens with adjuvant groups were significantly 4-fold greater than the unadjuvanted groups (*P* < 0.05), indicating that the adjuvant enhanced immune responses. The titers of IgG antibody induced by A35, B6, and M1 were comparable.

Similarly, mice immunized with Mix + A, which produced the highest neutralizing antibody titers of 2560, had a statistically significant neutralizing response compared to the PBS group (GMT = 903 versus GMT = 10, *P* < 0.05). Interestingly, the neutralizing antibody titers induced by M1 + A group (GMT = 557) were slightly lower than that of the Mix + A group (GMT = 903), and made statistical differences compared to the PBS group (*P* < 0.05). In addition, vaccination with the A29 antigen could also produce a certain level of neutralizing antibodies against the MVA regardless adding adjuvant. In contrast, neutralizing antibodies induced by other protective antigens were lower (Fig. 4B). The results indicated that vaccinations with M1 or A29 antigens developed cross-neutralizing antibodies against the MVA, which could thus be further developed as prominent antigen-based vaccines for the orthologous orthopoxvirus, such as mRNA or recombinant protein vaccines.

Overall, MVA proved highly efficacious and elicited MPXV antigen-specific humoral and cellular immune responses. Moreover, MPXV PAs also induced cross-neutralizing antibodies against the MVA to activate humoral immune responses; indicating that MVA developed robust cross-reactive antibody responses to the predominant PAs of the recent Mpox and neutralized the MPXV effectively, which might offer protection against MPXV infection.

## Discussion

In light of a continuous increase in Mpox, ring vaccination has been implemented to halt the potential spread of Mpox globally. In the present work, we elucidated that the MVA could produce specific IgG antibodies to various MPXV PAs (A29, M1, H3, I1, A35, and B6), along with neutralizing antibodies against the MVA and MPXV. Moreover, it also elicited cellular immune responses against A29, I1, and B6 antigens. Further analysis revealed that the major recombinant PAs could induce cross-neutralizing antibodies against the MVA, indicating that MVA induced robust cross-reactive immune responses against the present Mpox and might contribute to protection against disseminated MPXV infection.

The recent Mpox outbreaks result from genetic changes in MPXV, which are caused by lineage B.1 of the West African clade (MPXV Clade 3). This lineage exists at least 46 single-nucleotide polymorphisms, including 24 non-synonymous single-nucleotide polymorphisms [13, 31]. Notably, the insertion and deletion mutations in the DNA of the MPXV strain may be responsible for the current Mpox outbreak [32]. Genes of orthopoxvirus were highly conserved in the central region, and the genomes of MPXV and VARV shared a high level of sequence similarity (96.3%). The amino acid sequence of viral particle protein encoded by MPXV shared 91.7–99.2% similarity with VARV [33]. MVA is a vaccinia virus that passaged more than 570 in CEF, during which about 15% of the genome was deleted and mutated [34]. In this study, the predominant PAs of interest (A29, M1, H3, I1, A35, and B6) were at least 93% identical to the present MPXV and MVA strains evaluated. Given the high levels of sequence homology among the PAs from MVA and MPXV, understanding whether MVA can provide a protective immune response to the current MPXV remains a research concern.

MVA vaccine has been shown to induce binding antibodies against L1, A33, and B5, as well as specific T-cell responses, protecting against MPXV [30]. Other researchers also suggested that the vaccinia virus Tiantan strain yielded antibodies cross-reactive with MPXV PAs in immunized mice [35]. Our study revealed that, besides M1, A35, and B6 antigens, MVA induced specific antibodies against MPXV A29, H3, and I1 antigens by activating humoral immune responses. In particular, the inductions of cross-neutralizing antibody response against MVA were also elicited by MPXV PAs, especially the mixed PAs. Moreover, the PAs adjuvanted with AddaVax, which was a squalene-based oil-in-water nanoemulsion based on MF59 and could activate Th1/Th2 responses [36], provided more cross-reactivity to the MVA than those non-adjuvant, indicating that the AddaVax adjuvant had an immuno-enhancing effect in stimulating neutralizing antibodies. Similar studies found that A27 and B5 proteins with an adjuvant-induced neutralizing antibody responses against the vaccinia virus and provided complete protection [18]. Importantly, in the present study, it was notable that MVA vaccination induced both MVA-neutralizing and MPXV-neutralizing antibodies. Particularly, the MPXV-neutralizing antibodies induced by 10^5.7^ TCID_50_ MVA (10^7^ TCID_50_/mL, 50µL/dose) in our PRNT with 600 pfu of MPXV, might be comparable to those induced by 10^6^ pfu MVA with 100 pfu of Wyeth [37], while the latter could protect immune-deficient mice in the Western Reserve challenge model. These data indicated MVA might neutralize the MPXV effectively and activate a broad range of humoral immune responses, which might protect against the recent MPXV, probably for emergency use as a Mpox vaccine.

T lymphocytes are important in controlling orthopoxviral infections. Studies have shown that MVA immunization could rapidly activate CD8^+^ T-cell-mediated protective immunity against lethal ECTV and VACV infections [38, 39]. High levels of IFN-γ-producing CD8^+^ and CD4^+^ T cells were detected following VACV immunization [40]. In the current work, when we tested whether inoculations of MVA could induce T-cell-mediated immune response, MVA was sufficient to produce A29, I1, and B6-specific cytokines secreting splenocytes, probably mainly belonging to CD8^+^ and CD4^+^ T cells. The cytokines (IFN-γ, TNF-α, IL-4, IL-17A, and IL-22) were somehow enhanced by MPXV PAs stimulations after MVA immunization. Possibly, the activation of MVA-specific cytokines might be efficient in the immune response. Interestingly, we found that IL-17A, and IL-22 secreted by Th17 cells respond strongly to the stimulation. Briefly, Th17 T cells might be able to help with B cell proliferation, antibody production, and class switching [41]. It has been demonstrated that Th17 cells could mediate protection against *Mycobacterium tuberculosis* infection, probably by promoting CD4^+^ T cell recruitment to pulmonary sites of infection and accelerating pathogen clearance [42]. Another study also reported that the modified MVA encoding mycobacterial proteins enhanced numbers of CD4^+^ and CD8^+^ T cells producing IL-17 in the lung mucosae, including IL-17, IL-2, and IFN-γ, as well as markedly reducing *Mycobacterium tuberculosis* titers recovered from pulmonary tissues after challenge [43]. Therefore, numerous cytokines might involve in MVA-mediated protection.

Furthermore, this research indicated that vaccinations with A29 and M1 antigens could neutralize the MVA. In contrast, vaccinations with A35, B6, H3, and I1 antigens failed to neutralize the MVA, even though these four antigens could induce MVA-specific IgG binding antibodies, probably due to their neutralizing abilities slightly lower than A29 and M1 antigens. It may be unclear about the role of neutralizing antibodies as a correlate of protection against disease and transmissibility [44]. These data are also consistent with the findings that the A35 antibody failed to neutralize EV *in vitro*; the protectivity mediated by the A35 protein probably involved a mechanism different from simple antibody binding [23, 45]. Notably, A29 was important for virus replication, regulating cell entry and virus egress, among which antibodies binding to A29_21 − 40_ epitope neutralized mature viruses in a complement-dependent manner and provided protection [46]. We demonstrated that vaccination with A29 elicited cross-binding and cross-neutralizing activity for the MVA, which activated A29-specific T-cell response. Such a robust and diverse neutralizing antibody response and cellular immune response likely explain that A29 is the predominant antigen target for evaluating the immunogenicity of the MVA vaccine or novel vaccines such as recombinant protein vaccine and mRNA vaccine. In addition, similar to other studies [47], MVA induced high-level M1-specific IgG antibody responses, whereas M1 vaccination elicited neutralizing antibody against MVA, underlying M1, which is important for viral entry into cells and MV attachment or penetration, especially M1_25 − 34_ and M1_113 − 131_ epitopes, can mediate neutralizing antibody in protective responses [48]. M1 could be considered one of the antigen targets designed for Mpox vaccines. The resulting data revealed that MVA contributed significantly to cross-react with MPXV PAs and neutralized MPXV *in vitro*. Nevertheless, it is not well understood whether MVA can protect against MPXV in live MPXV animal challenge models and humans, and this remains a key future research need.

In summary, the findings presented here revealed that MVA exhibited superiority in humoral and cellular immune responses against various MPXV PAs, importantly neutralized MVA and MPXV, meanwhile, PAs elicited cross-reactive antibody-mediated to the MVA, suggesting MVA might be suited as an emergency candidate vaccine for the current Mpox. Remarkably, MPXV protective antigens A29 and M1 can be considered major antigen targets for novel orthologous orthopoxvirus vaccine such as recombinant protein or mRNA vaccine.

## Data Availability

All data generated or analysed during this study are included in this published article.
